# Supplementary material for: Comprehensive Analysis of Biomass from Chlorella sorokiniana Cultivated with Industrial Flue Gas as the Carbon Source
Source: Molecules. 2024 Jul 18;29(14):3368. doi: 10.3390/molecules29143368 (PMC11279589; doi:10.3390/molecules29143368)
Supplement: Supplementary file 1 [file molecules-29-03368-s001.zip › molecules-3076671-supplementary.pdf]

SUPPLEMENATRY DOCUMENTS

Comprehensive Analysis of Biomass from *Chlorella sorokiniana* Cultivated with Industrial Flue Gas as the Carbon Source

Arjun H. Banskota, Roumiana Stefanova, Joseph P. M. Hui, Tessa Bermarija, Kevin Stemmler, Patrick J. McGinn and Stephen J. B. O'Leary*

Aquatic and Crop Resource Development Research Centre, National Research Council Canada, 1411 Oxford Street, Halifax, Nova Scotia, B3H 3Z1, Canada

*Correspondence: arjun.banskota@nrc-cnrc.gc.ca; Tel.: 1-902 426 7372

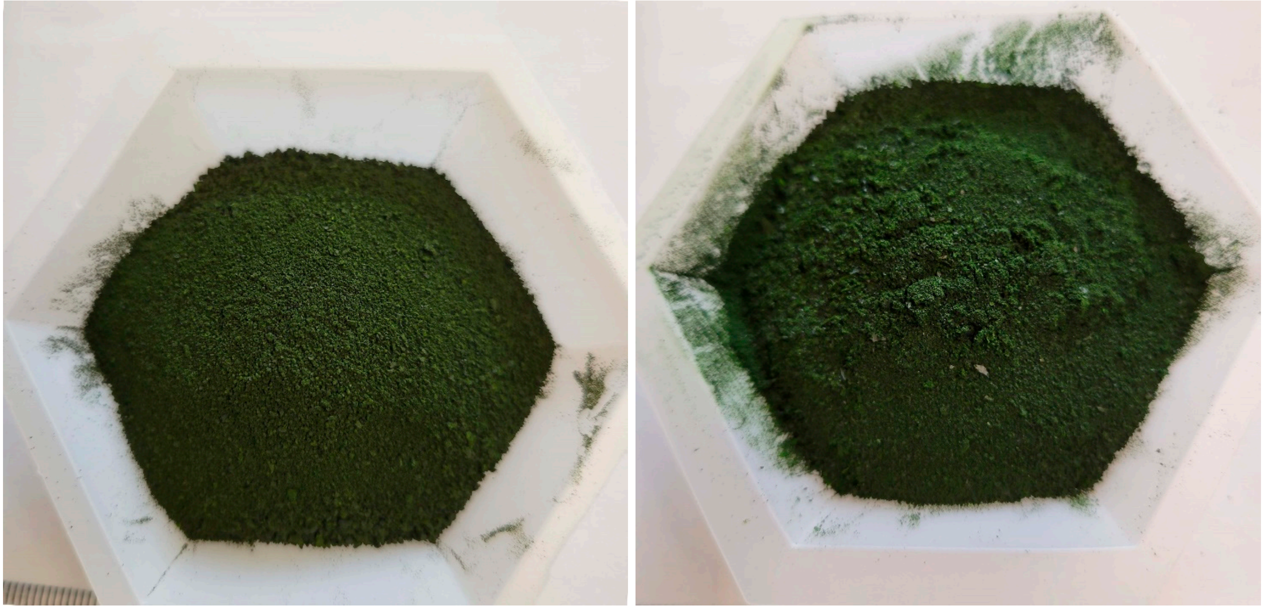

Figure S1. Freeze-dried algal biomass of *C. sorokiniana* cultured in 20 L (left) and 25000 L Photobioreactors (right).

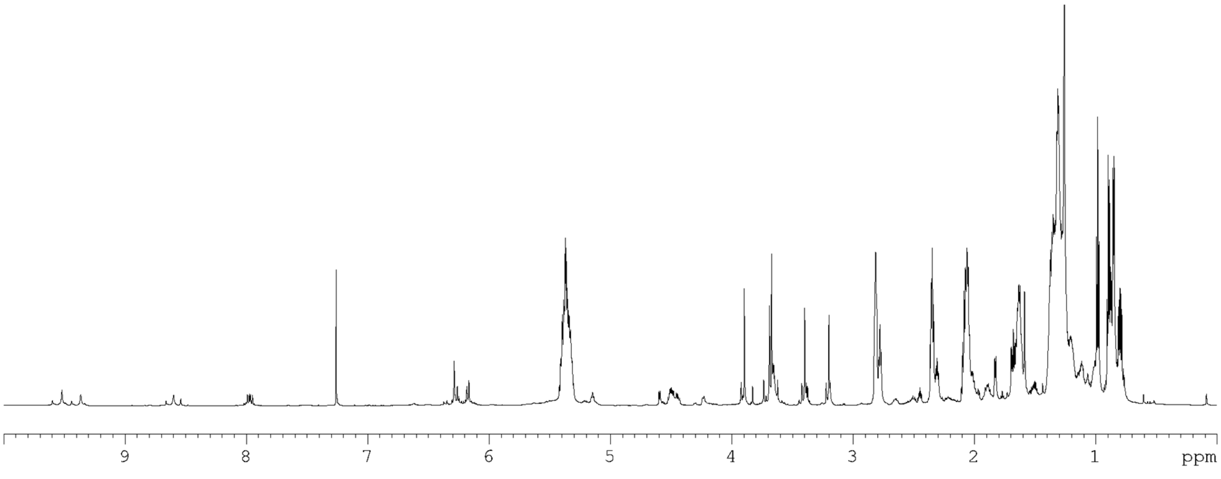

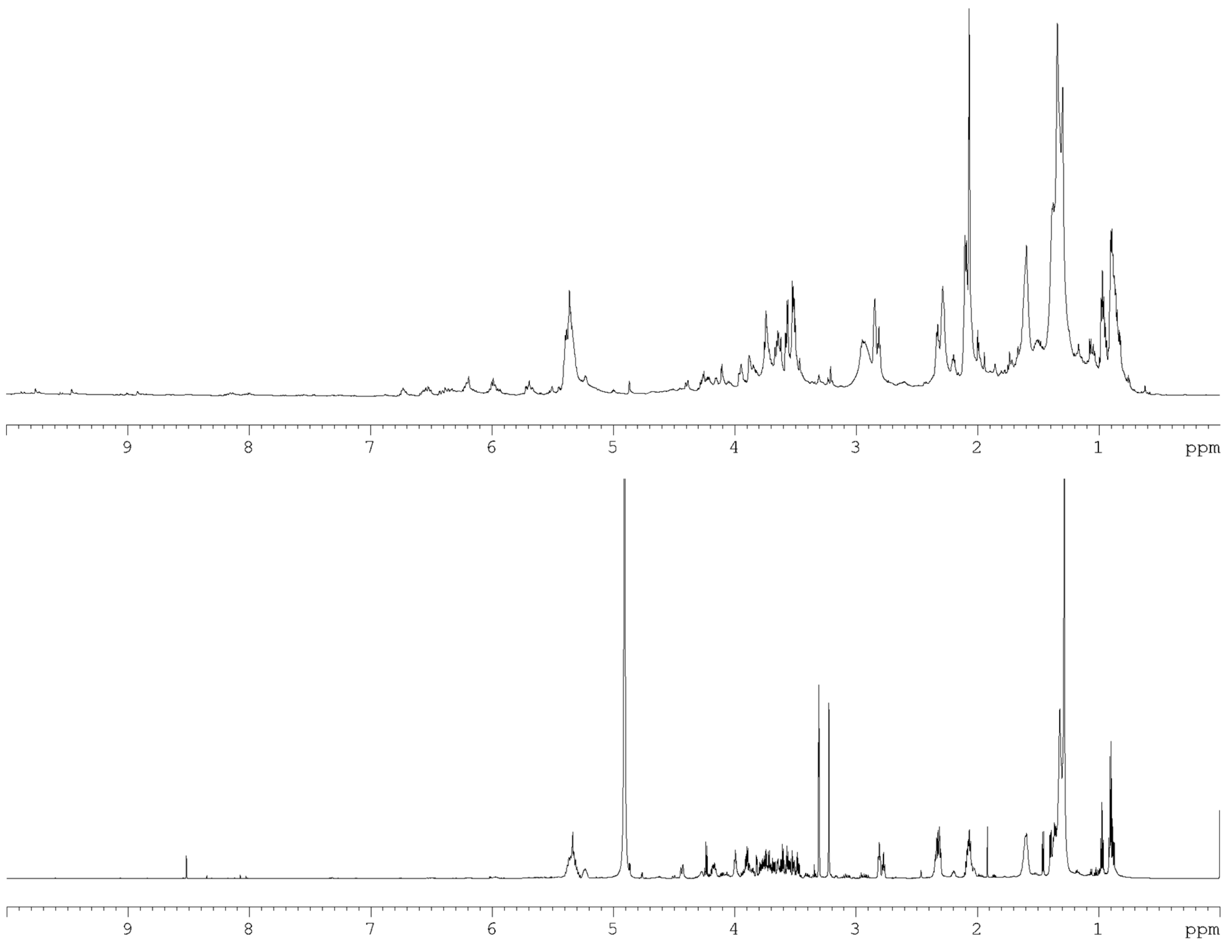

Figure S2. Proton NMR spectrum of [A] neutral lipid (top), glycolipid (middle) and phospholipid (bottom) fractions from lipid extract from *C. sorokiniana* cultured in a 25000 L Photobioreactor.

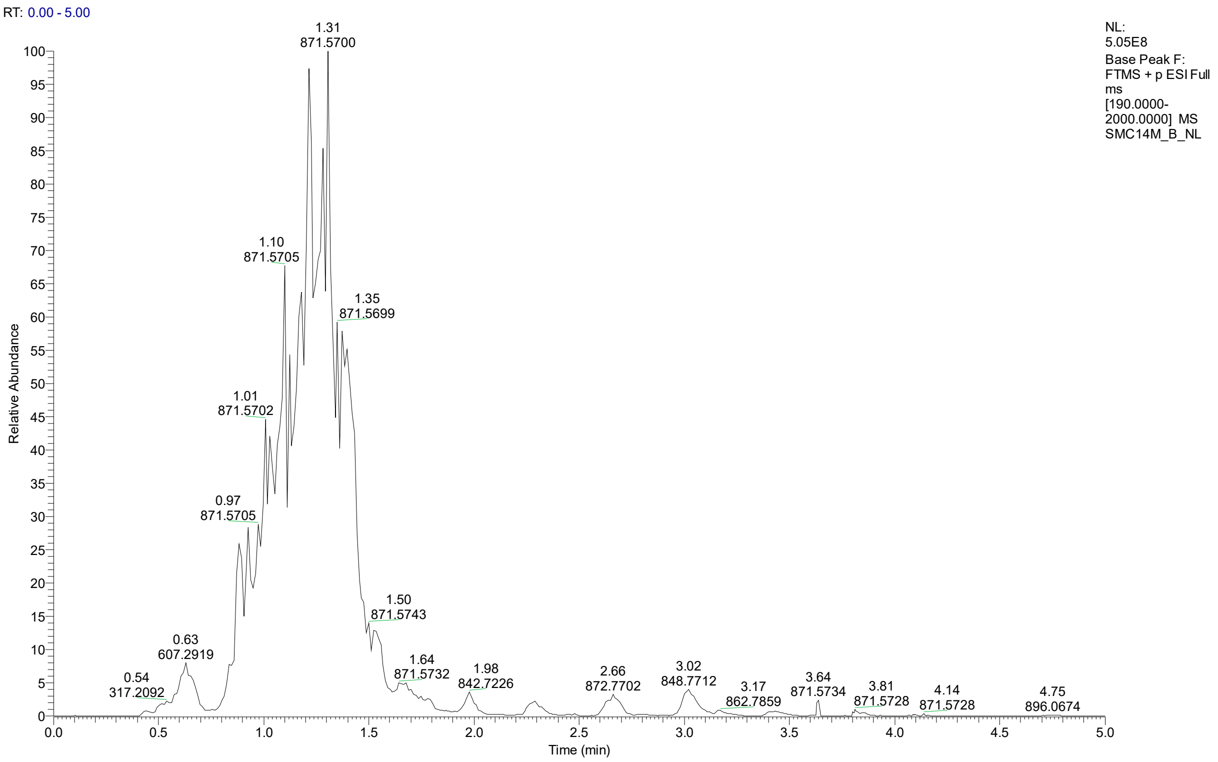

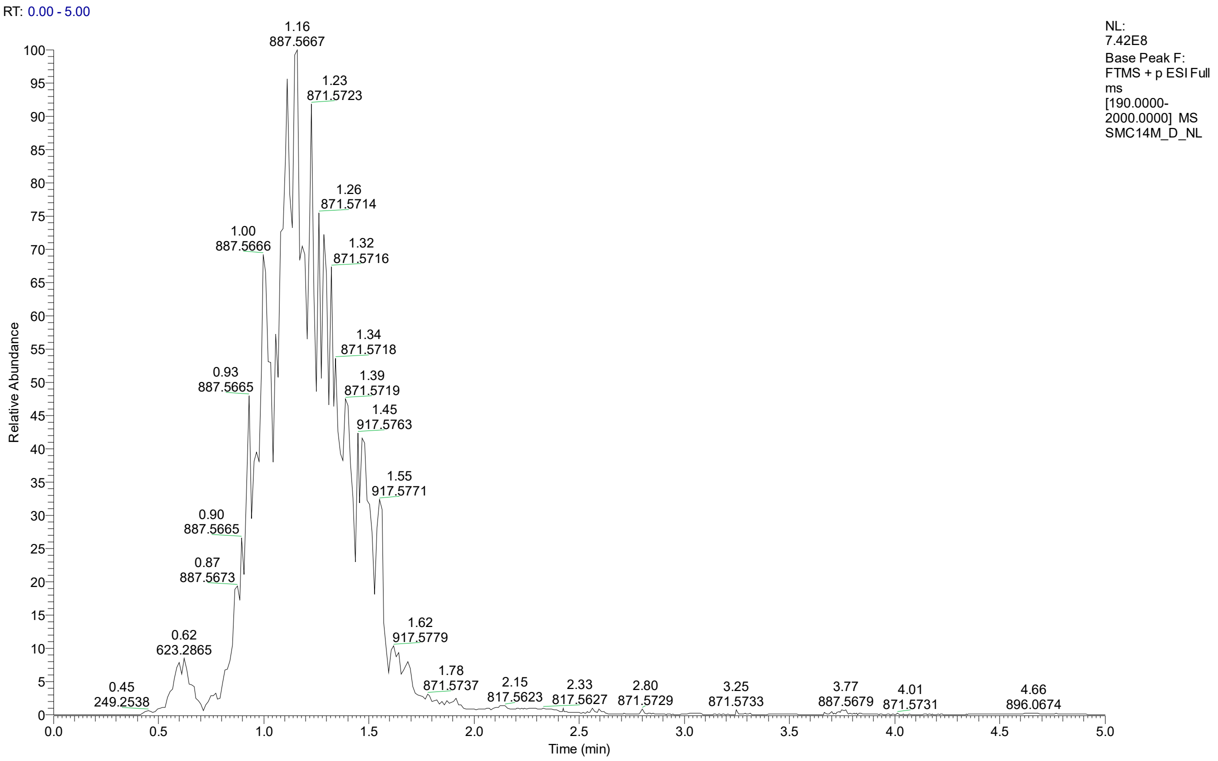

Figure S3. Total ion chromatograms (TICs) of the neutral lipid fraction of *C. sorokiniana* biomass cultured in 20 L (top) and 25000 L (bottom) Photobioreactors.

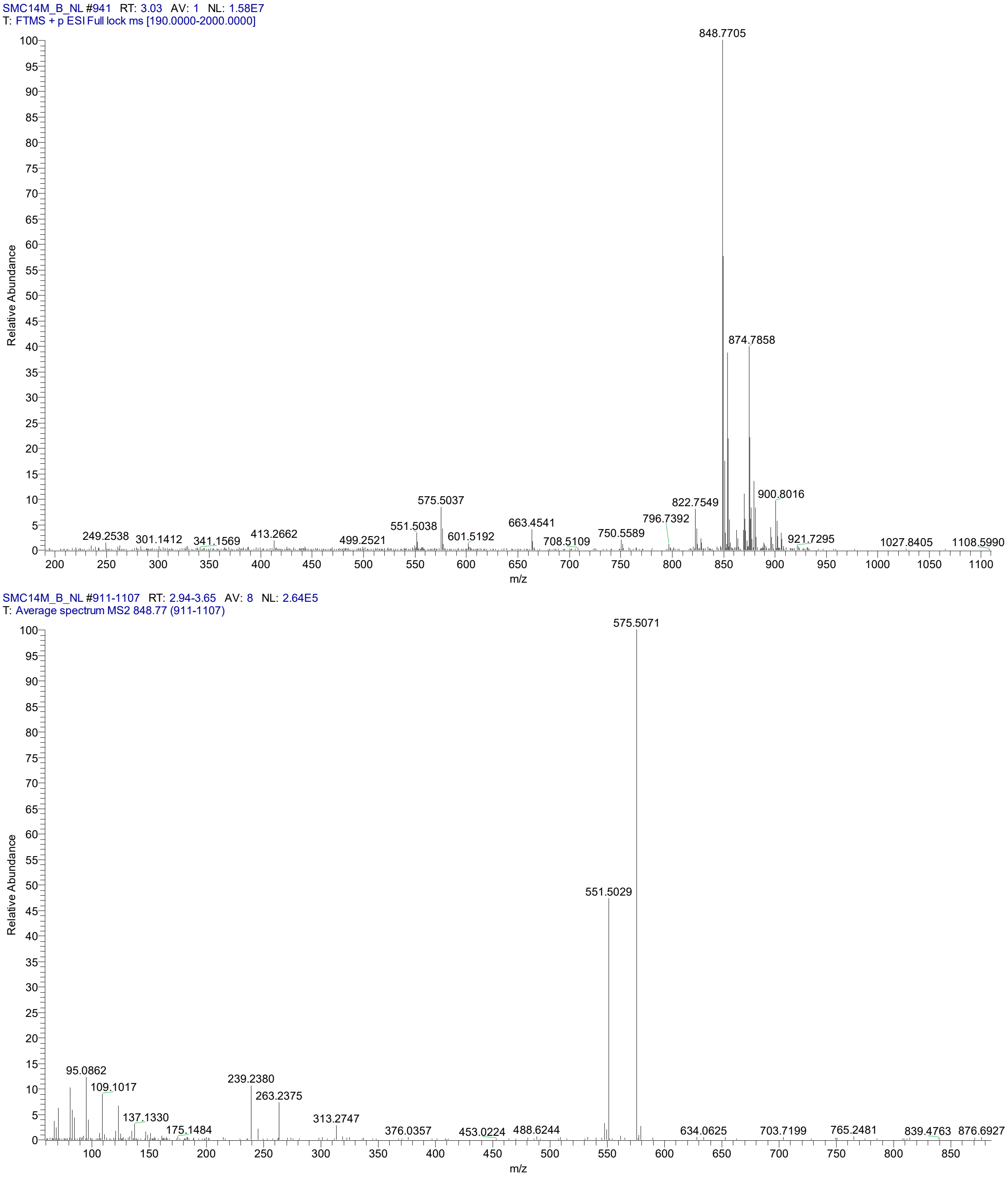

Figure S4. Mass spectrum of metabolites eluted at 3.03 min (top) and fragmentation ions corresponding to neutral loss of TAG (50:2).

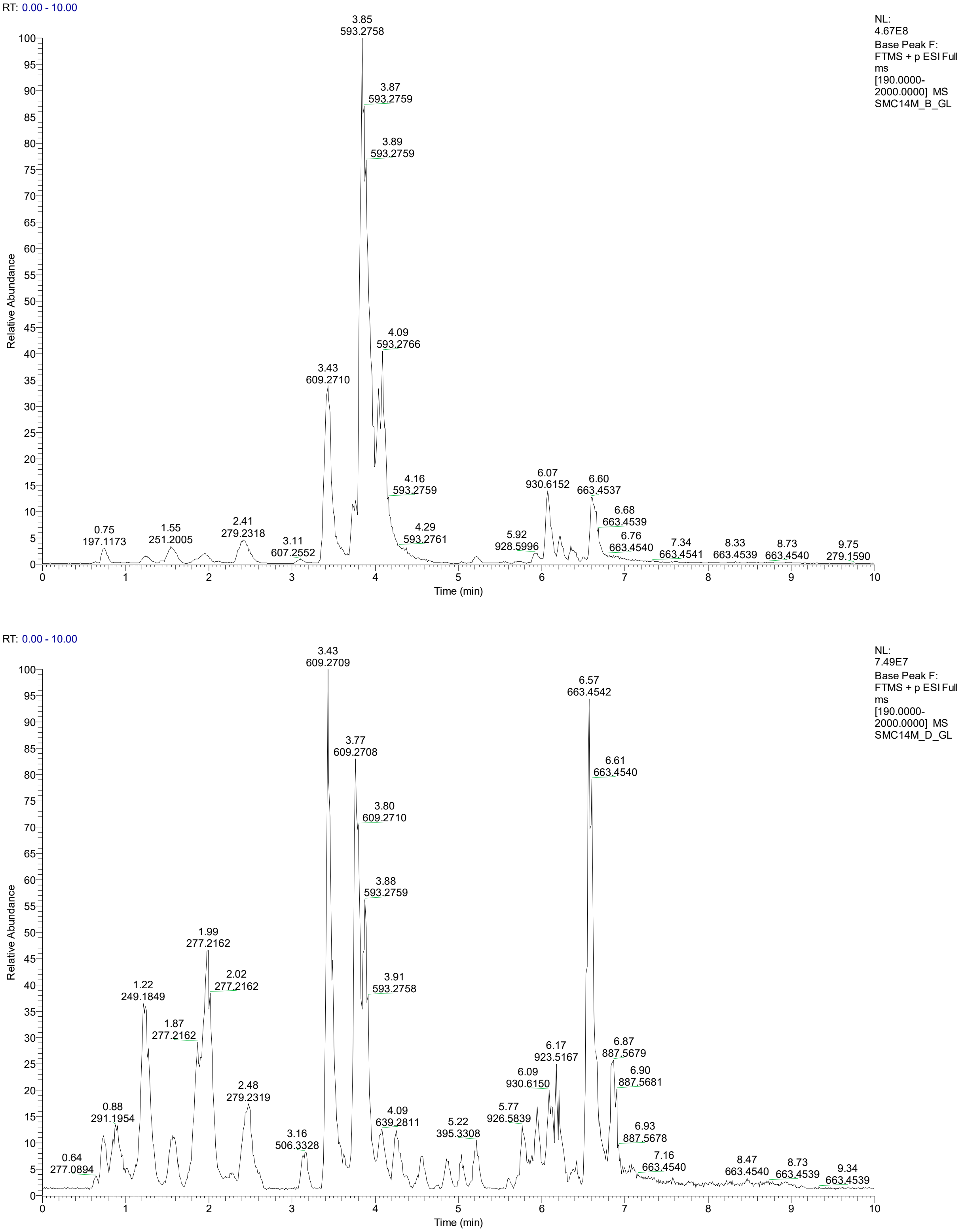

Figure S5. Total ion chromatograms (TICs) of the glycolipid fraction of *C. sorokiniana* biomass cultured in 20 L (top) and 25000 L (bottom) Photobioreactors.

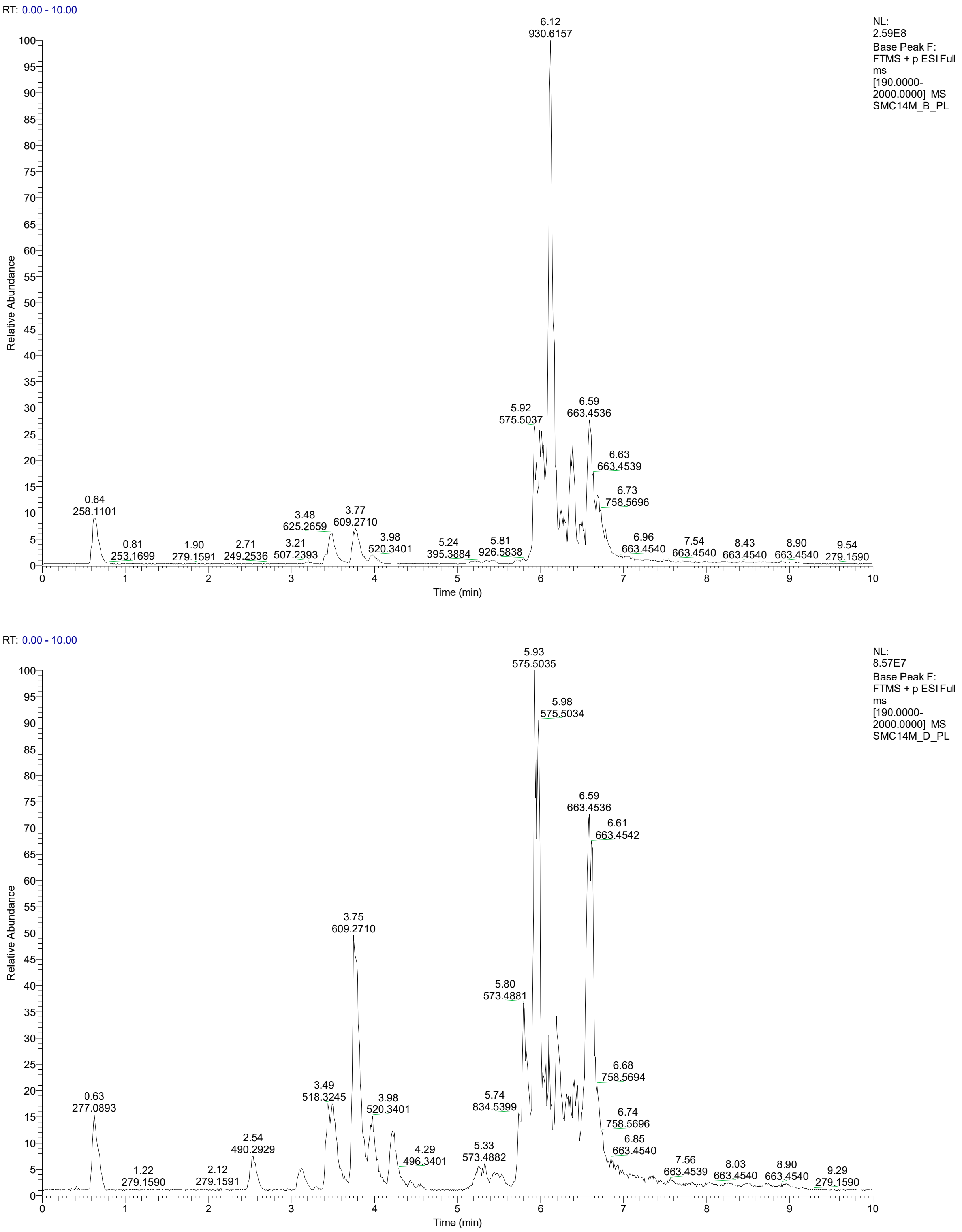

Figure S6. Total ion chromatograms (TICs) of the phospholipid fraction of *C. sorokiniana* biomass cultured in 20 L (top) and 25000 L (bottom) Photobioreactors.

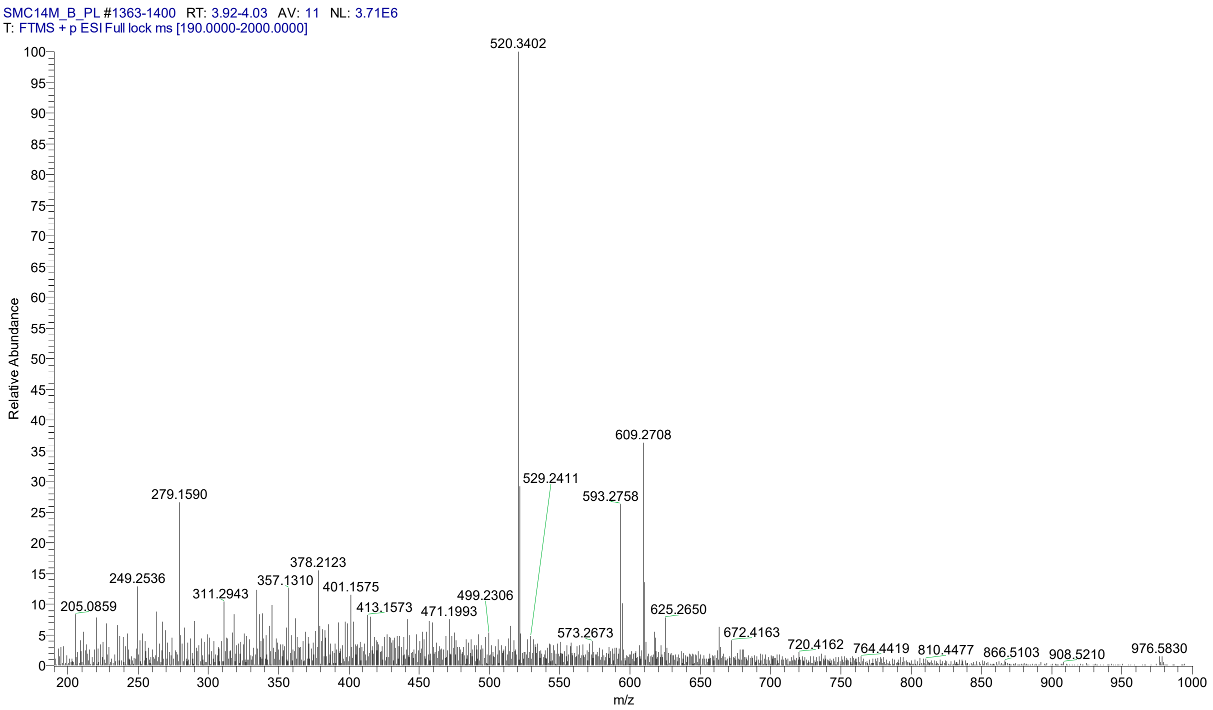

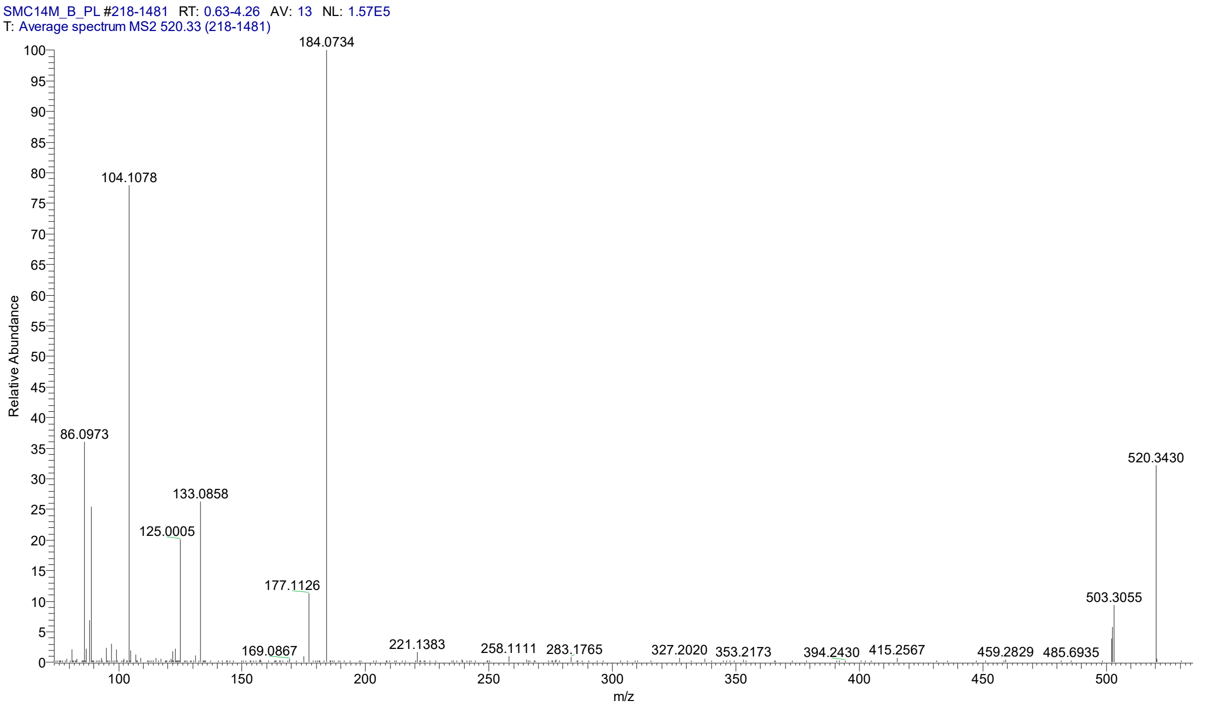

Figure S7. Mass spectrum of metabolites eluted between 3.92-4.00 min (top) and fragmentation ions corresponding to LPC (18:2).

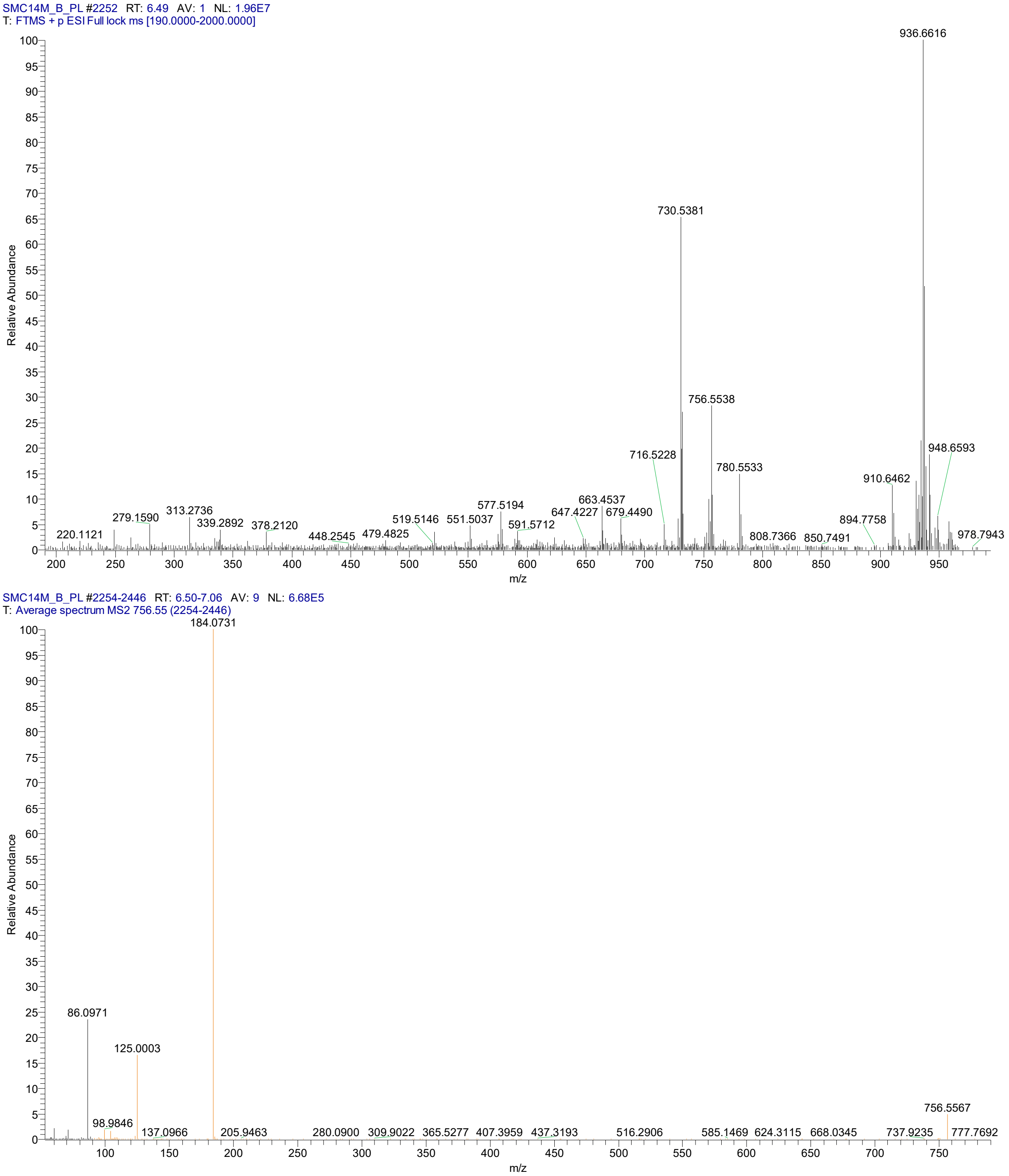

Figure S8. Mass spectrum of metabolites eluted at 6.49 min (top) and fragmentation ions corresponding to PC (34:3) in positive mode (bottom).

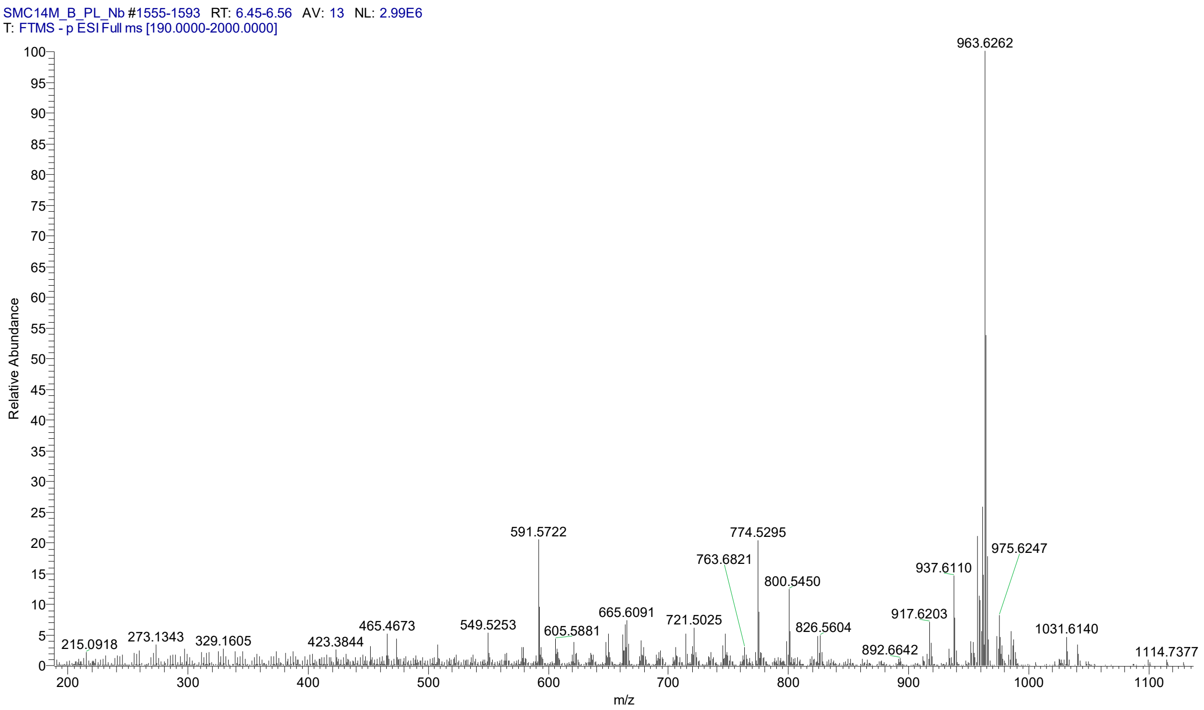

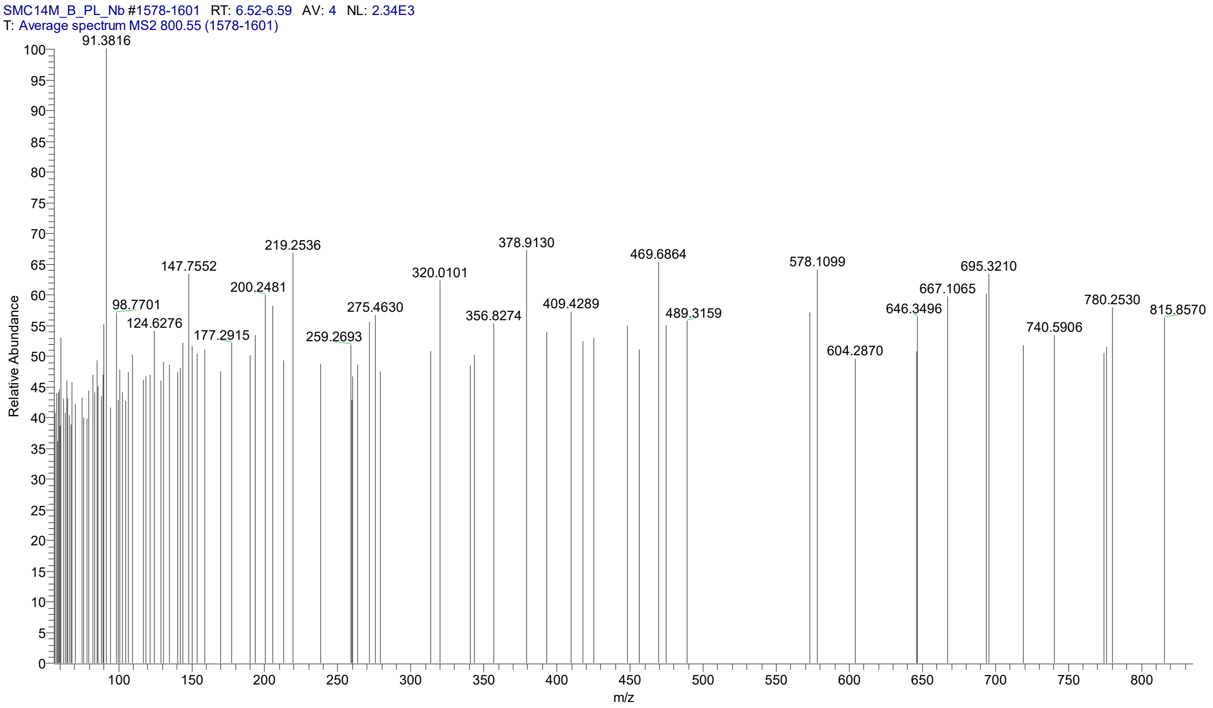

Figure S9. Mass spectrum of metabolites eluted between 6.45-6.56min (top) and fragmentation ions corresponding to PC (34:3) in negative mode (bottom).

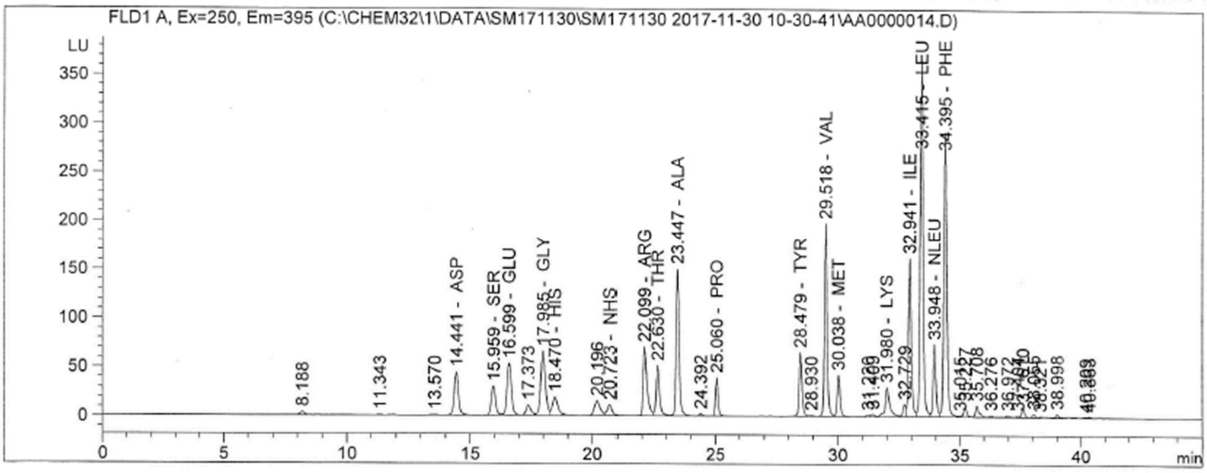

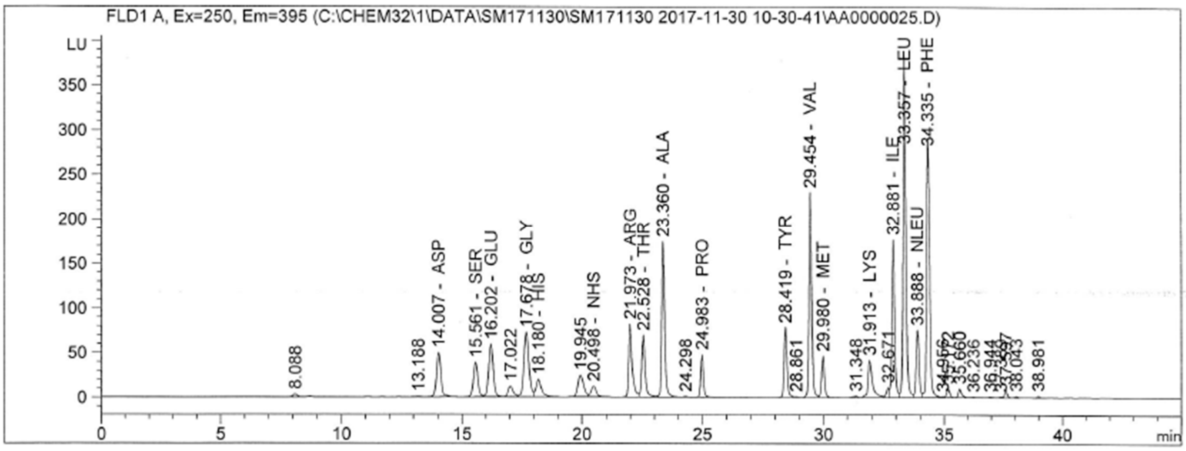

Figure S10. HPLC chromatogram of amino acid analysis of protein isolate extracted from biomasses collected from 20 L (top) and 25000 L photobioreactors (bottom).

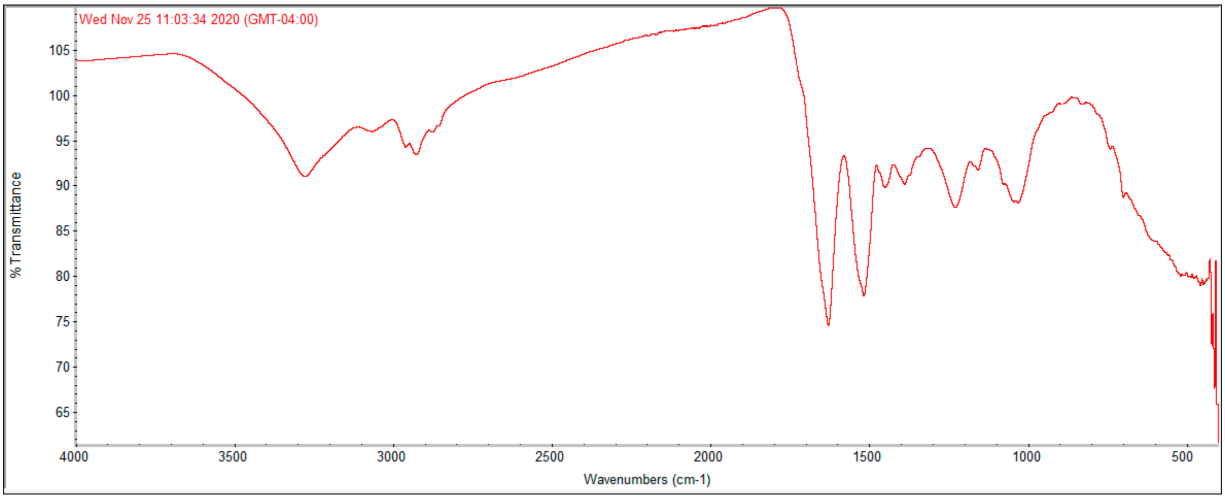

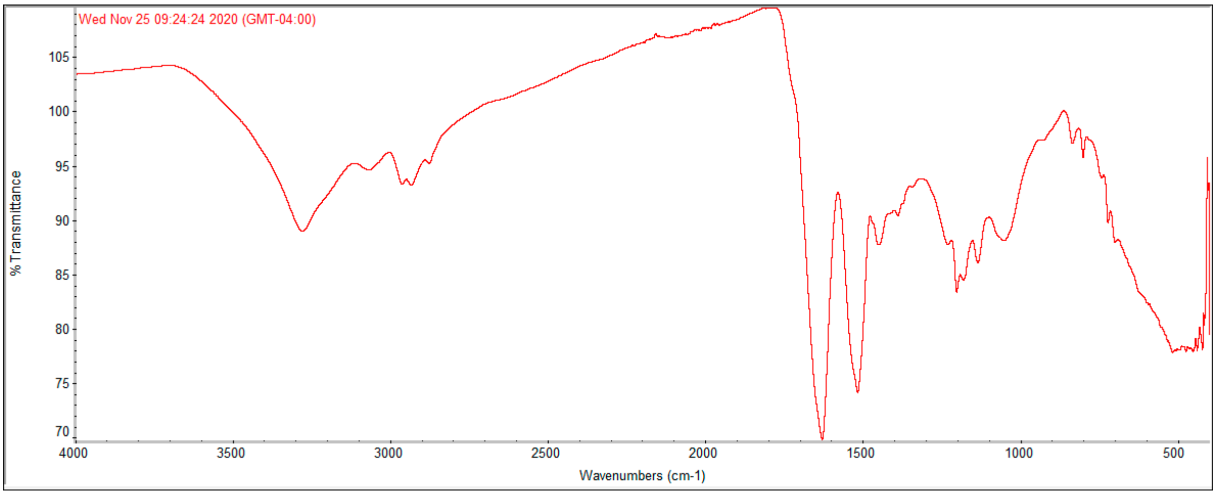

Figure S11. IR spectra of protein isolates extracted from residual biomass of 20 L (top) and 25000 L photobioreactors (bottom).

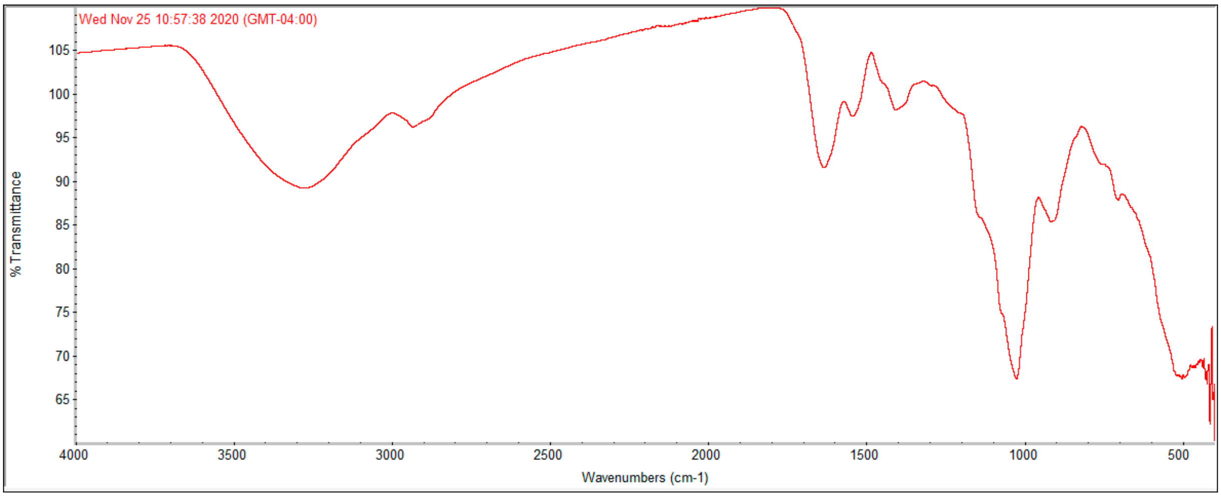

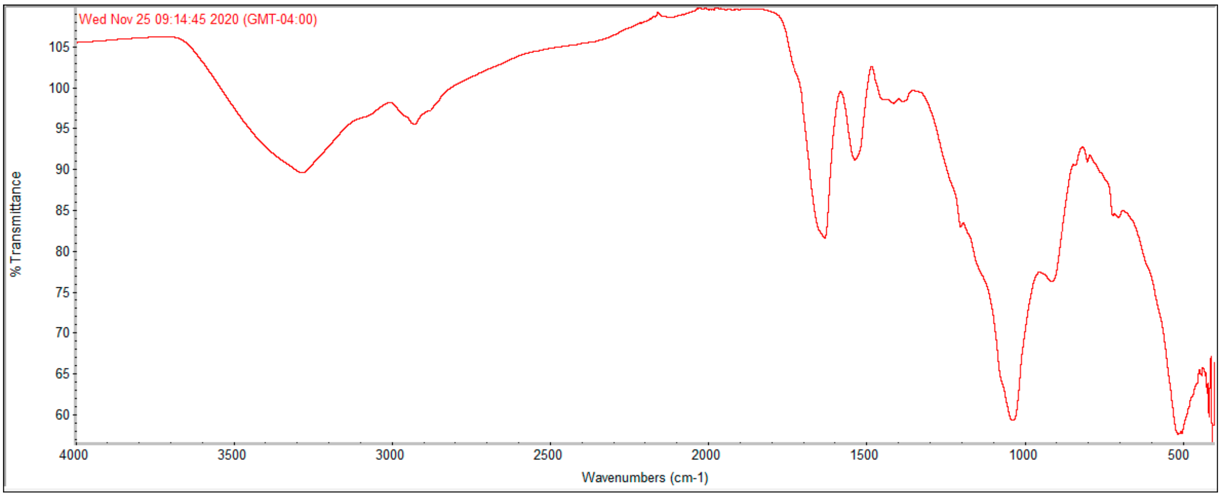

Figure S12. IR spectra of polysaccharides extracted from residual biomass of 20 L (top) and 25000 L photobioreactors (bottom).

Table S1. Fatty acid profile of total lipid extracts. Results are expressed in mg/g of total lipid and the percentage of individual fatty acid in the oil is given in the parenthesis.

Fatty acid	*20 L Photobioreactor*	*25000 L Photobioreactor*
Myristic acid	1.2±0.2 (0.4)	-
Myristoleic acid	6.0±1.0 (2.2)	6.5±0.6 (3.0)
Pentadecanoic acid	0.5±0.4 (0.2)	1.4±0.5 (0.6)
cis-10-Pentadecenoic acid	-	-
Palmitic acid	76.5±4.0 (27.5)	55.4±1.7 (25.1)
Palmitoleic acid	3.3±0.1 (1.2)	3.9±0.3 (1.8)
C16:2 n-4	0.5±0.1 (0.2)	0.2±0.3 (0.1)
Heptadecanoic acid	0.7±0.1 (0.2)	1.2±0.2 (0.5)
16:3 n-4	1.3±0.0 (0.5)	1.5±0.2 (0.7)
cis-10-Heptadecenoic acid	-	-
C16:4 n-1	1.1±0.1 (0.4)	1.9±0.1 (0.9)
Stearic acid	2.9±0.1 (1.1)	1.6±0.0 (0.7)
Oleic acid	20.3±0.7 (7.3)	4.4±0.1 (2.0)
C18:1 n-7	3.3±0.1 (1.2)	-
Linoleic acid	124.5±4.2 (44.8)	65.2±1.7 (29.6)
C18:2 n-4	0.3±0.1±0.1 (0.1)	0.2±0.0 (0.1)
r-linolenic acid	-	0.2±0.0 (0.1)
C18:3 n-4	-	0.1±0.1 (0.0)
a-linolenic acid	24.5±0.8 (8.8)	69.9±1.7 (31.7)
C18:4 n-3	0.3±0.1 (0.1)	0.2±0.0 (0.1)
C18:4 n-1	0.8±0.1 (0.3)	0.7±0.0 (0.3)
Arachidic acid	0.3±0.1 (0.1)	0.2±0.0 (0.1)
cis-11-Eicosenoic acid	1.9±0.1 (0.7)	1.1±0.0 (0.5)
cis-11,14-Eicosadienoic acid	0.5±0.2 (0.2)	0.4±0.0 (0.2)
cis-8,11,14-Eicosatrienoic acid	-	-
Henicosanoic acid	-	-
Arachidonic acid	-	-
cis-8,11,14-Eicosatrienoic acid	-	-
C20:4 n-3	-	-
Eicosapentaenoic acid	1.8±0.1 (0.7)	1.1±0.0 (0.5)
Behenic acid	0.9±0.3 (0.3)	0.4±0.0 (0.2)
Erucic acid	3.4±0.2 (1.2)	2.0±0.1 (0.9)
Docosadienoic acid	-	-
C22:4 n-6	-	-
Lignoceric acid	0.4±0.1 (0.2)	0.4±0.0 (0.2)
Docosahexaenoic acid/C24:1 n-9	0.7±0.1 (0.2)	0.4±0.0 (0.2)
SFA	**83.4±5.0 (30.0)**	**60.6±2.4 (27.5)**
MUFA	**38.8±2.0 (14.0)**	**18.4±1.0 (8.3)**
PUFA	**155.7±5.1 (56.0)**	**141.5±3.6 (64.2)**
Total FA mg/g (%)	**277.9±11.9 (100)**	**220.5±7.0 (100)**

- not detected, Fatty acid analysis was done on triplicate.

Table S2. Separation of $CHCl_3$/MeOH extract obtained from biomass harvested from a 25000 L photobioreactor into fractions and identification of metabolites within each fraction

Fraction	Eluent (mL)	Weight (g)	Metabolites identified by HPLC or LC/MS analysis
**1**	$CHCl_3$ (400)	0.238	β-carotene<br>α-carotene<br>fatty acid-16:2<br>fatty acid-16:3<br>fatty acid-18:2 n-6)<br>fatty acid-18:3
**2**	$CHCl_3$ (300)	0.830	pheophytin-a*<br>fatty acid-16:2<br>fatty acid-16:3<br>fatty acid-18:2<br>fatty acid-18:3
**3**	$CHCl_3$ (1600)<br>$CHCl_3$/MeOH (19:1, 100)	0.467	Lutein<br>chlorophyll-a<br>chlorophyll-b<br>fatty acid-16:2<br>fatty acid-16:3<br>fatty acid-18:2<br>fatty acid-18:3
**4**	$CHCl_3$/MeOH (19:1 – 4:1, 1700)	1.190	MGDG *m/z* 740.4<br>MGDG *m/z* 766.4<br>MGDG *m/z* 738.4<br>MGDG *m/z* 764.4<br>MGDG *m/z* 792.4<br>MGDG *m/z* 742.4<br>MGDG *m/z* 768.4<br>MGDG *m/z* 762.4<br>MGDG *m/z* 794.5<br>DGDG *m/z* 928.5<br>DGDG *m/z* 930.5<br>DGDG *m/z* 932.5

* Degraded product of chlorophyll-a

The freeze dry biomass (15.0 g) collected from the pilot-scale photoreactor was extracted with chloroform/MeOH (1:1, 100 ml x 3) by sonicating (15 min) at room temperature. The combined extract was then dried under vacuum yielding chloroform/MeOH extract (3.10 g). The chloroform/MeOH extract was further portioned into five fractions by Flash chromatography using a silica gel column (12 x 4.5 cm) eluting with chloroform/MeOH gradient [Chloroform 400 mL-Fraction 1, 0.238 g; chloroform 300 mL – Fraction 2, 0.830 g; chloroform 1600 mL – Fraction 3, 0.410; chloroform/MeOH (19:1) 100 ml – Fraction 4, 0.057 g; chloroform/MeOH (19:1-5:1) 1700 mL – Fraction 5, 1.191 g]. The major compound present within the fractions were identified either by HPLC and LC/MS with single quadrupole ms detector.
